# Necessity and Effect of Combating *Legionella pneumophila* in Municipal Shower Systems

**DOI:** 10.1371/journal.pone.0114331

**Published:** 2014-12-09

**Authors:** Ragnhild Wiik, Anne Vatland Krøvel

**Affiliations:** IRIS International Research Institute of Stavanger, Stavanger, Norway; Purdue University, United States of America

## Abstract

The objective was to obtain research-based, holistic knowledge about necessity and effect of practiced measures against *L. pneumophila* in municipal shower systems in Stavanger, Norway. The effects of hot water treatment and membrane-filtering were investigated and compared to no intervention at all. The studies were done under real-world conditions. Additionally, a surveillance pilot study of municipal showers in Stavanger was performed. The validity of high total plate count (TPC) as an indication of *L. pneumophila* was evaluated. A simplified method, named “dripping method”, for detection and quantification of *L. pneumophila* was developed. The sensitivity of the dripping method is 5 colony-forming units of *L. pneumophila*/ml. The transference of *L. pneumophila* from shower water to aerosols was studied. Interviews and observational studies among the stakeholders were done in order to identify patterns of communication and behavior in a *Legionella* risk perspective. No substantial effects of the measures against *L. pneumophila* were demonstrated, except for a distally placed membrane filter. No significant positive correlation between TPC and *L. pneumophila* concentrations were found. *L. pneumophila* serogroup 2–14 was demonstrated in 21% of the 29 buildings tested in the surveillance pilot. Relatively few cells of *L*. *pneumophila* were transferred from shower water to aerosols. Anxiety appeared as the major driving force in the risk governance of *Legionella*. In conclusion, the risk of acquiring Legionnaires' disease from municipal shower systems is evaluated as low and uncertain. By eliminating ineffective approaches, targeted *Legionella* risk governance can be practiced. Risk management by surveillance is evaluated as appropriate.

## Introduction

Extensive research has been done on *Legionella pneumophila* and other *Legionella* species. In spite of this, the handling of *L. pneumophila* in general, and in shower systems in particular, may be characterized by the word “uncertainty” [1], [2], [3]. This uncertainty concerns pathogenicity/virulence, host susceptibility, prevention methods, infection sources, and how to execute and analyze water samples.

*L. pneumophila* was named after being recognized as the causative agent of pneumonia among American legionnaires during a convention in 1976. The hotel's air condition system was identified as the infection source. The genus *Legionella* is primarily associated with Legionnaires' disease [4]. About 50 different species of *Legionella* have been identified [1, 5, 6 7, 8]. The species *L. pneumophila* is found to account for at least 90% of reported cases of Legionnaires' disease [7], [9]. So far, at least 15 serogroups (sg) of *L. pneumophila* have been identified [5], [7], [10]. Only a few of the approximately 15 serogroups of *L. pneumophila* have been associated with disease [5], [9], [10], [11]. In Europe, *L. pneumophila* sg 1 has been attributed to more than 85% of the cases Legionnaires' disease [9]. Sg 1 is further divided into types which seem to represent different levels of virulence [11], [12], [13]. In summary, a few subgroups of *L. pneumophila* sg 1 appear to be the source of most cases of Legionnaires' disease.

*L. pneumophila* is an opportunistic pathogen concerning Legionnaires' disease [4], [14]. Statistics show that the most susceptible individuals are male smokers over 40 years of age who are also suffering from diseases like diabetes or chronic heart disease [1], [9]. It has been reported that less than 1% of the individuals exposed to *Legionella* develop Legionnaires' disease [15]. Different degrees of mortality have been reported for individuals suffering from Legionnaires' disease, i.e. from 0.1 to 30% [16], [17].

In Stavanger, Norway, the first, and so far the only known case of contracting Legionnaires' disease from municipal showers, occurred in 2007 in an indoor swimming pool facility [3]. Prior to this event, there were no municipal procedures aimed at reducing the risk of being infected by *L. pneumophila* from showering. Based on the Norwegian guidelines for the prevention of *Legionella* infection [18], [19], [20], a routine of temperature control, quarterly chlorination of shower heads, and monthly hot water treatment of all municipal shower systems was implemented in Stavanger municipality. High total heterotrophic plate count (TPC) was used as an indication of the presence of *L. pneumophila.* Initially, a high TPC was defined as a concentration above 1000 colony forming units/ml (cfu/ml). At TPC exceeding 1000 cfu/ml, a second hot water treatment was initiated. The consequence was a comparatively high and demanding flushing frequency. A new limit of 10 000 cfu/ml was therefore introduced after half a year. The limit of 10 000 cfu/ml was in accordance the “EWGLI Technical Guidelines for Investigation, Control and Prevention of Travel Associated Legionnaires' Disease” concerning cooling towers [21]. We are not acquainted with research data, however, that support that a TPC higher than 10 000 cfu/ml is a valid indicator of the presence of *L. pneumophila*.

The lack of knowledge mentioned above may be ascribed to a fragmentary research approach concerning the genus *Legionella*. By “fragmentary” we mean that only one or a few perspectives have been studied at a time. Additionally, evaluation of prevention measures is often case-oriented [1], [22], [23] which makes it difficult to generalize the results. The conclusion from comprehensive literature studies in which different measures against *L. pneumophila* have been compared, is that no single method stands out as unambiguously effective [1], [24], [25], [26], [27].

The uncertainty about the risk associated with *Legionella* may easily lead to formation of myths [2], [3]. Myths appear like “truths” and are thereby seldom questioned. One such myth seems to be that all kinds of man-made aerosol-producing water systems with temperatures in the range of 25–45°C have identical potential of inflicting Legionnaires' disease. In Norway, this has resulted in implementation of laborious measures towards *L. pneumophila* in various kinds of man-made aerosol-producing water systems [3], [19]. However, there is scant knowledge about the relative risk associated with different water sources; i.e. cooling towers, air scrubbers, showers, whirlpools, spas and public fountains [28], [29], [30]. In Norway, outbreaks of community-acquired Legionnaires' disease have exclusively been associated with outdoor systems, i.e. cooling towers and air scrubbers [18], [19], [20]. A second and related myth seems to be that the risk of being infected by *L. pneumophila* from man-made aerosol-producing water systems is considerable. Extensive use of the concept “outbreak” in relation to Legionnaires' disease [10], [28] may contribute to such a myth. An outbreak has been defined as two or more cases where the onset of illness is closely linked in time and space and where there is suspicion of, or evidence of, a common source of infection [31]. Colloquially, the outbreak concept indicates that a substantial number of individuals are infected from the same agent more or less simultaneously. The concept may be appropriate when it comes to Legionnaires' disease from cooling towers, air scrubbers and air condition systems as well as for Pontiac fever [19]. When it comes to shower systems, however, the incidents seem to be sporadic. The sporadic nature of community-acquired legionellosis in general is supported by Fisman et al. [29], who found that 94% of 240 cases over 9 years were sporadic.

*L. pneumophila* is known to be transferred to humans via anthropogenic aerosols [32]. According to our knowledge, the size of the *Legionella*-containing aerosols has seldom been discussed as a variable influencing the risk of being infected. Schoen and Ashbolt [32], however, represent an exception with their inclusion of aerosol size as a variable in modeling *Legionella* exposure during showering events. The risk should increase with decreased aerosol size [33], [34]. Generally, smaller droplets remain airborne for longer, and aerosols of 5 µm in diameter or less penetrate deep into the lungs [33], [34]. However, larger droplets can diminish in size by evaporation and still contain the initial number of bacteria. In spite of being aerosol-generating, municipal showers in Norway have only once been identified as the source of Legionnaires' disease [3]. This may have something to do both with the size of the water droplets of the shower water and the relative humidity (RH) in the shower room. It has been demonstrated that transmission of influenza virus depends on the relative humidity of the air; with low RH promoting disease [33].

After having practiced nationally recommended hot water treatment towards *L. pneumophila* in shower systems over a period of two years, representatives of Stavanger municipality, Norway, questioned if this measure had the intentional effect. The objective of the present study was to gain research-based, interdisciplinary knowledge about necessity and effect of combating *L. pneumophila* in municipal shower systems. In the effect studies, we focused on hot water treatment and filtration.

## Materials and Methods

### Investigative design

The research project consisted of five complementary parts. The first part concerned the effect of measures against *L. pneumophila* in municipal shower systems. Our studies were mainly performed under real-world conditions and only exceptionally in the laboratory. One school and three architectonically identical nursing homes were selected for the *in situ* experiments. In the second part, approximately 10% of the municipal building mass containing shower systems was selected for a surveillance pilot study. The showers of these buildings were all thermally treated according to municipal routines. The third part consisted of a longitudinal study of the effect of thermal treatment of showers in sports complexes, measured as total bacterial plate counts and presence of *L. pneumophila*. Parts 2 and 3 provide supplementary data to the case studies in the first part. In the fourth part transfer of *L. pneumophila* from shower water to aerosols was investigated. The last part consisted of qualitative interviews and observations of governmental and non-governmental stakeholders. While the three first parts of the project concern the effects of measures against *Legionella*, parts 4 and 5 also concern the necessity of combating *Legionella*. The combination of microbial and psychological studies made it possible to investigate *Legionella* as a public health issue in municipal water systems in a more holistic way.

All the research described in this article was approved by IRIS and Stavanger municipality, Norway. The research has been performed according to scientific ethical norms.

### Municipal buildings

Stavanger municipality permitted access to all the locations included in the studies and approved the investigative design of the project.

The investigation of effect of measures against *L. pneumophila* was performed in municipal buildings having comprehensive shower systems. The schools and sports complexes had shower rooms, each with 5 to 12 showers. In other cases there was one shower per bath room as in nursing homes and wardrobes of teachers and instructors. One of the main experimental buildings was a two-storied nursing home consisting of three identical wings. This building is designated Nursing Home 1. Two additional nursing homes in Stavanger municipality, architectonically identical to Nursing Home 1 were also included. They were designated Nursing Home 2 and 3. The three nursing homes were built in the period of 1985–1988 and had practically identical heating, ventilation and sanitation systems. These buildings made it possible to perform nearly controlled experiments under real-world conditions. Regular hot water treatment was not performed at Nursing Home 1. Some individual showers, however, were occasionally hot water-treated in order to study the effect on the total bacterial plate counts. For nearly one year, a membrane filter was placed in one of the wings, while the other two wings were not treated at all. The shower systems in Nursing Homes 2 and 3 were regularly hot water-treated during the experimental period of four years. The hot and cold water was mixed in each individual shower armature.

Another main experimental building was a school completed in 1988, designated School 1. The shower system of this school had just been completely renovated at the onset of the study in 2008. The system consisted of 3 water heaters connected in series. The hot and cold water was centrally mixed to a water temperature of 38°C. A circulation pipe of 80 m ensured the maintenance of 38°C throughout the shower system. Our studies were focused on a shower room having 9 showers. Only a few days after completion of the new shower system, i.e. in 2008, a membrane filter (see “Measures against *L. pneumophila*”) was installed in the water flow after the point of mixing hot and cold water. After studying the effect of the filter for a period of about nine months, the filter was detached. Thereafter the shower system was out of operation for about three months due to a combination of summer holidays and renovation of the wardrobes associated with shower rooms. *L. pneumophila* was not observed anywhere in the system during the filtration period. Three months after demounting the membrane filter at School 1, *L. pneumophila* was demonstrated in the water from the showers. Since then, *L. pneumophila* has been present in the shower system (from 2009 to so far 2014). This has made it possible to perform real-world experiments with respect to *L. pneumophila* for several years.

### The surveillance pilot study

In addition to the experimental buildings described above, a surveillance project was initiated in 2010 in order to get an indication of the frequency of *L. pneumophila* in municipal shower systems. Twenty nine buildings out of about 250 were included in this pilot survey. The building categories were nursing homes, kindergartens, office buildings, schools (primary and junior secondary), and sports complexes. Water samples were taken from representative showers in each building. The sampling frequency was every 6 months.

### Measures against *L. pneumophila*

#### Hot water treatment

Hot water treatment was performed according to the instructions of Stavanger municipality which were based on the guidelines of the Norwegian Institute of Public Health [18], [19]. Each shower system with respective pipelines and armatures should be flushed at least monthly for 5 minutes with hot water holding at least 65°C.

#### Proximal filtration

Two different system filters and one armature filter were tested. The system filters were placed in the pipeline system upstream of the shower rooms. System filters from Enwa AS and Aquanor AS were installed at Nursing Home 1 and School 1, respectively.

At Nursing Home 1, the water of one of the three wings of the building was filtrated by the membrane filter from Enwa AS. The water supply of the other two wings constituted untreated control groups. The filter was tested from August 2008 to December 2009.

The system filter at School 1 was tested during a nine month period from autumn 2008 to the onset of the summer season in 2009. Simultaneously as mounting the system filter, a rough filter (pores of 5 µm) was mounted at the water inlet of the building. Faucets were mounted before and after the rough filter as well as before and after the system filter (membrane filter). Water from the faucets and showers was sampled from several times a week to once every second week depending on the stability of the TPCs.

#### Distal filtration

An antilegionella armature from Shelby Teknikk AS was installed at School 1 one year after the first demonstration of *L. pneumophila* in the shower system. This shower armature includes a membrane filter with pore size of 0.02 µm and a hydraulic valve. When the shower is in use, water runs through the filter which retains bacteria from the water jet. The hydraulic valve regulates the alternation between filtration of the water and draining of retained bacteria through an opening at the bottom of the armature.

The purpose of this experiment was to test if the filter has the potential of preventing *L. pneumophila* from penetrating to the shower water. Water was regularly sampled from the Shelby shower for three years. We sampled both from the shower water and the non-filtrated drainage water descending at the bottom of the armature. The drainage water from the Shelby shower was designated “compact water” in order to pin point that it is not forming a substantial amount of aerosols. The results were compared to results of regular showers of the same shower system with respect to TPCs and *L. pneumophila*.

All the filters had automatic cleaning devices. Filters from Enwa AS and Shelby Teknikk AS were cleaned by reversed water flush. The pore sizes of the filters were 0.02–0.03 µm.

### Sampling and bacterial analyses

Before the initiation of the present research project, 250 ml of water were sampled from the individual shower for bacterial analysis. The sample of 250 ml constituted a mix of water from the shower armature and the adjacent pipelines. This mixing made it impossible to decide if there was a substantial difference between bacterial concentration and composition in different parts of the system. Therefore, the sampling regime was changed from collecting one single sample of 250 ml to collecting at least two samples á 50 ml at intervals of about 30 seconds. For press button showers, the samples were taken from the onset of each pulse. The samples were designated first, second, third water etc. The first water sample represented the status of the shower head/armature and was taken at the onset of the shower. Except for the first water, the water samples represented the water present in the pipeline system, designated system water. For traditional showers, the mixing battery was set to ordinary showering temperature, i.e. 37–40°C, before the start of sampling. Both press button showers and traditional ones had standard armatures, i.e. not water saving armatures. The flow of the press button showers was approximately 10 l per minute. The 50 ml sampling tubes were ray-sterilized. Samples were processed at our laboratory within 2 hours. The water samples were analyzed with respect to TPC and *L. pneumophila*.

#### Analysis of TPC

TPC was determined according to the standard “ISO 6222∶1999(E)” [35]. In order to simplify the traditional spread plate method, we used the drop plate method concerning the water samples [36], [37]. The individual Water Plate Count agar plate was divided into sectors according to number of dilutions. Three parallels of 10 µl each were dripped on each sector. The samples were diluted up to 100 times by the use of distilled water. The plates were incubated at 37°C for about 48 hours. The detection limit of the method is 100 cfu/ml.

#### Analysis of *L. pneumophil*

A revised method based on the standard “ISO 11731∶1998(E)” [38] was developed in order to simplify the procedure. The standard method was simplified by omitting the centrifugation or filtration step. The same agar media as described in the ISO standard [38] were applied. Standard products from Oxoid (CM0655, SR01010C, and SR0152E) were used to produce the GVPC (Glycin, Vancomycin, Polymyxin B and Cycloheximidine) agar plates selective for *Legionella* species in environmental samples. The agar plates were prepared in house since we experienced the *L. pneumophila* colonies as easier to recognize on home-made plates as compared to the ones prepared by the manufacturer Oxoid. This is in accordance with the observations of McCabe et al. [39].

Three parallels from each water sample were analyzed with respect to *L. pneumophila*. One single agar plate was divided into three sectors and 200 µl of undiluted water sample was dripped from a micropipette on each segment resulting in 15–20 droplets. Each droplet was allowed to spread naturally. The plates were left upright on the laminar hood with the lids partly off to dry and thereafter incubated at 37°C for 3–5 days. For other *Legionella* species up to 10 days of incubation is recommended. The number of colonies per parallel was counted, and the mean value per ml was calculated. The detection limit for *L. pneumophila* was 5 cfu/ml. In need of higher sensitivity, the water volume of the parallels has to be increased. One single *Legionella* plate can easily hold 1 ml of water. Our dripping plate method is based on the drop plate method [36], [37] and is similar to the method of Leoni and Lagnani [40]. The main difference is that each parallel of the dripping plate method is based on several drops while only one in the drop plate method.

In some cases, i.e. when testing the efficiency of the Shelby filter, we increased the sensitivity of our analyses. One liter of water was filtrated through a filter of pore size 0.22 µm. The bacteria on the filter were suspended in 5 ml of sterile water. In theory, this implies a 200 fold increase of bacterial concentration. By measuring TPCs, it was recognized that the real concentration was increased only 50–100 fold which indicates that only 25–50 per cent of the bacteria loosened from the filter or was cultivatable after filtration.

Presumptive *L. pneumophila* colonies were transferred by streaking onto plates without L-cystein, serotyped by agglutination test (Oxoid) and finally identified by sequence-based typing (SBT) at the National Reference Laboratory for *Legionella* in Norway [41], [42], [43]. The confirmative results were reported as *L. pneumophila* sg 1 or the omnibus designation sg 2–14. The strain *L. pneumophila* ATCC 33152 was used as a phenotypic reference with respect to colony and cell morphology in the identification process.

Swab samples were taken from the surface of e.g. water pipes, inside of mixing batteries and/or from shower tubing using sterile cotton swabs. The swabs were immediately swabbed over agar plates prepared for TPCs and *L. pneumophila*, respectively. The incubation conditions were as described above. The swab samples gave an impression of the extent and complexity of the biofilm.

#### Determination of *L. pneumophila* in air

Air samples were taken from shower rooms in order to investigate the extent of *L. pneumophila* in aerosols from showers known to contain this bacterium. Also TPC of the air samples was determined. The air sampler “SAS Super 90” combined with plates containing the same solidified media as for the water samples was used [44]. The volume of air sampled was standardized to 500 l. Sampling was performed (i) before turning on the showers, (ii) while the water was running, (iii) after turning off the showers, and (iv) outdoor. The air samples were taken from the breathing zone of a person of approximately 1.65 m height.

The validity of the air-sampling method was evaluated by analyzing a known aerosolized sample of 0.9 ml with a known concentration of *L. pneumophila*. A plastic cylinder of 30 cm length was mounted onto the lid of the sampler. The sample was sprayed into the cylinder using an aerosol spray can just after turning on the air sampler. The recovery of *L. pneumophila* was calculated by comparing the recovery from 100 and 500 l of air samples. One single spray of 0.9 ml was used for each individual measurement which was performed in triplicates. The bacterial solution of the aerosol can was prepared by transferring one colony of *L. pneumophila* from a GVPC plate to sterile water. Either colonies from original water samples or colonies passaged once were used. The plate had been incubated for 4 days. The dilutions were performed with the intention to get a reliable number of 25–300 colonies per plate.

### Longitudinal analyses of TPCs in municipal sports complexes

Through the years 2007–2009, 3714 water samples from the shower systems of all municipal sports complexes in Stavanger were analyzed with respect to TPC. These analyses were commissioned by Stavanger municipality and performed by Eurofins Scientific, Stavanger. The same culture medium and incubation conditions as described for TPC in the present paper were used by Eurofins. Samples were taken at least monthly from each system. We analyzed the TPCs by use of statistical methods (ANOVA and Scheffé's test) in order to test if the buildings could be separated into significant different groups with respect to general bacterial concentrations. Continued sampling from the sports complexes was done in 2010–2012. In the period 2007–2009, 250 ml samples were taken. From 2010 successive samples of 50 ml as described above were taken. After 2010, the samples were analyzed with respect to both TPC and *L. pneumophila*.

### Persistence and vulnerability of *L. pneumophila* under laboratory conditions

A piece of piping of 30 cm were cut off the pipeline system at School 1 and brought to the laboratory. One end of the piece was plugged. The biofilm which consisted of both *L. pneumophila* and several other bacterial species had been left intact. The plugged piece of pipe was filled with cold tap water from a laboratory faucet. The tap water was negative with respect to *L. pneumophila*. Rubber film was used as cover. The piece of piping was incubated at room temperature for twelve months. A water sample of 2 ml was taken every second month. After a year, the piece of piping was hot water treated for 5 minutes. The temperature of the water started at 68°C and finished at 62°C. The tube was once again filled with cold tap water, and water samples of 2 ml were taken at least twice a year for three years.

### Statistical analyses

Our quantitative data consisted of bacterial concentrations, i.e. TPCs and concentrations of *L. pneumophila*. The data was analyzed by SPSS (Statistical Package for Social Sciences) and GraphPad Prism 5.0 (Graph Software, Inc.). We used analyses as t-tests, Analysis of Variance (ANOVA) combined with Scheffé's test, and both Pearson and Spearman correlations. In cases where the data were far from normally distributed, non-parametric tests were used, e.g. Spearman's rank correlation.

### Qualitative interviews and observations

Semi-standardized, individual telephone interviews [45] were performed in order to investigate how operating personnel practiced hot water treatment and sampling. In the period November 2008 to February 2009, operating personnel at six sports complexes and two nursing homes, altogether eight interviewees, were interviewed. Each and all had extensive practical experience with hot water treatment. The questions concerned laboriousness and efficiency of the treatment as well as precautions with respect to their own health.

In the period 2007–2011, a combination of non-standardized interviews and observations [45] was performed among stakeholders such as governmental representatives, municipal employees, the public, private property owners and commercial water treatment companies. Among these were physicians, scientists, teachers and parents. The interviews and observations were focused on how different stakeholders communicate with respect to *Legionella*. Our goal was to identify substantial driving forces behind the *Legionella* risk governance. Notes were taken during and after meetings, seminars and conversations (included telephone conversations). All together, approximately 200 individuals were included in this study.

The data from interviews and observations were analyzed by grounded theory approach [46].

## Results

### The revised and simplified method for detection and enumeration of *L. pneumophila*

In this project a revised and simplified method for detection and enumeration of *L. pneumophila* based on the standard ISO 11731∶1998(E) [38] was developed. The main deviation from the standard is the removal of the concentration step. The centrifugation or filtration step has been shown to result in comparatively low and variable recovery of cultivatable bacteria from water samples [47], [48] and thereby reduced reliability of the results. Omitting the concentration step decreases sensitivity compared to the standard and might increase the chance of getting false negative results for samples with a low bacterial count. However, the detection limit of the revised method is 5 cfu/ml, and since this is within the recommended action limit suggested by the European Working Group for Legionella Infections [21], the sensitivity was evaluated as satisfactory for routine surveillance of environmental samples.

### TPC – an invalid indicator of *L. pneumophila*

In Norway high TPCs have been recommended as an indicator of *L. pneumophila* [18], [19]. Accordingly, TPCs have been the most used measure of *L. pneumophila* in Norway.

Our dripping method for detection and enumeration of *L. pneumophila* has made it convenient to investigate the correlation between concentration of *L. pneumophila* and TPC. These two measures were systematically recorded for one year at School 1 which had *L. pneumophila* sg 2-14 in the shower system. The correlations between concentration of *L. pneumophila* and TPC were investigated both for the first water and system water, respectively. The correlations between these bacterial measures were very low and usually far from significant (Table 1). There was a weak, but significant, negative correlation between the concentration of *L. pneumophila* and TPC after thermal treatment (Table 1).

**Table 1:** Correlation between TPC and *L. pneumophila* (*L. pn.*) concentration (cfu/ml) in the first water and system water under ordinary conditions and after hot water treatment, respectively.

		First water		System water	
		*L. pn.*	TPC	*L. pn.*	TPC
*L. pn.* (cfu/ml) Ord. cond.^a^	Correlation Coefficient	1.000	−0.148	1.000	−0.053
	Sig. (2-tailed)		0.120		0.609
	N^c^	112	112	97	97
TPC (cfu/ml) Ord. cond.^a^	Correlation Coefficient	−0.148	1.000	−0.053	1.000
	Sig. (2-tailed)	0.120		0.609	
	N^c^	112	112	97	97
*L. pn.* (cfu/ml) Hot water^b^	Correlation Coefficient	1.000	−0.234*	1.000	−0.363^**^
	Sig. (2-tailed)		0.011		0.001
	N^c^	118	118	87	87
TPC (cfu/ml) Hot water^b^	Correlation Coefficient	−0.234*	1.000	−0.363^**^	1.000
	Sig. (2-tailed)	0.011		0.001	
	N^c^	118	118	87	87

*Correlation is significant at the 0.05 level (2-tailed). **Correlation is significant at the 0.01 level (2-tailed).

a Ordinary conditions.

b After hot water treatment.

c N: Number of samples.

**Figure 1:**
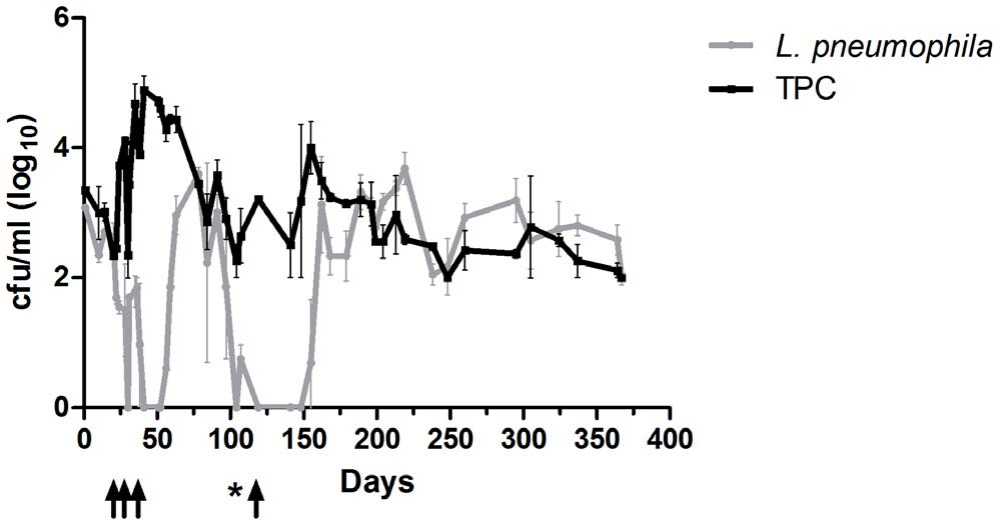
Correlation between *L. pneumophila* sg 2–14 and TPC in the shower system of School 1 over a period of 1 year. The arrows indicate time points for hot water treatment and the star represents a one-time chlorination treatment (Concentrate of di-n-decyl dimethylammonium chloride (<2%), quaternary ammonium combinations, benzyl-C8-18-alkyldimetyl, chlorides (<2%) and hexamethylene biguanide hydrochloride (<1%) (Barrier Tech Concentrate)) of the system.

The correlation studies at School 1 were performed when *L. pneumophila* was present in the system. In shower systems without detectable *L. pneumophila*, we have measured comparatively high TPCs (above 10^4^ cfu/ml) (Table 1, Fig. 1). The inference is that high TPCs did not indicate presence of cultivatable *L. pneumophila*.

**Figure 2:**
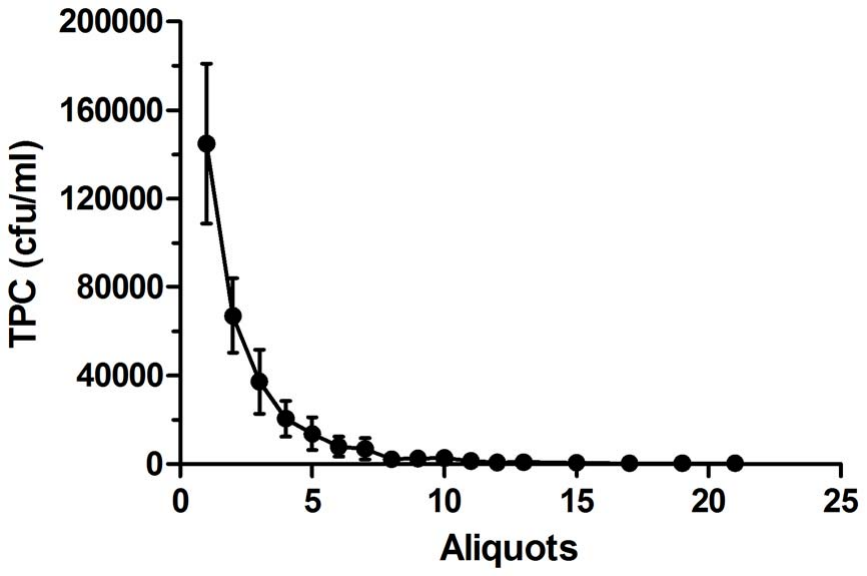
TPC as a function of number of aliquots. From the first aliquot of 50 ml after turning on a shower, the TPCs of successive water aliquots each of 50 ml rapidly decimated. The first 4 points were based on 6 showers. Points 5 and 6 were based on 5 and 6 showers, respectively. The remaining points were based on 2 to 6 showers (mean 3.6). The mean number of showers during the whole experiment was 4.2. The same set of showers was used through the whole experiment. The bars indicate SD.

**Figure 3:**
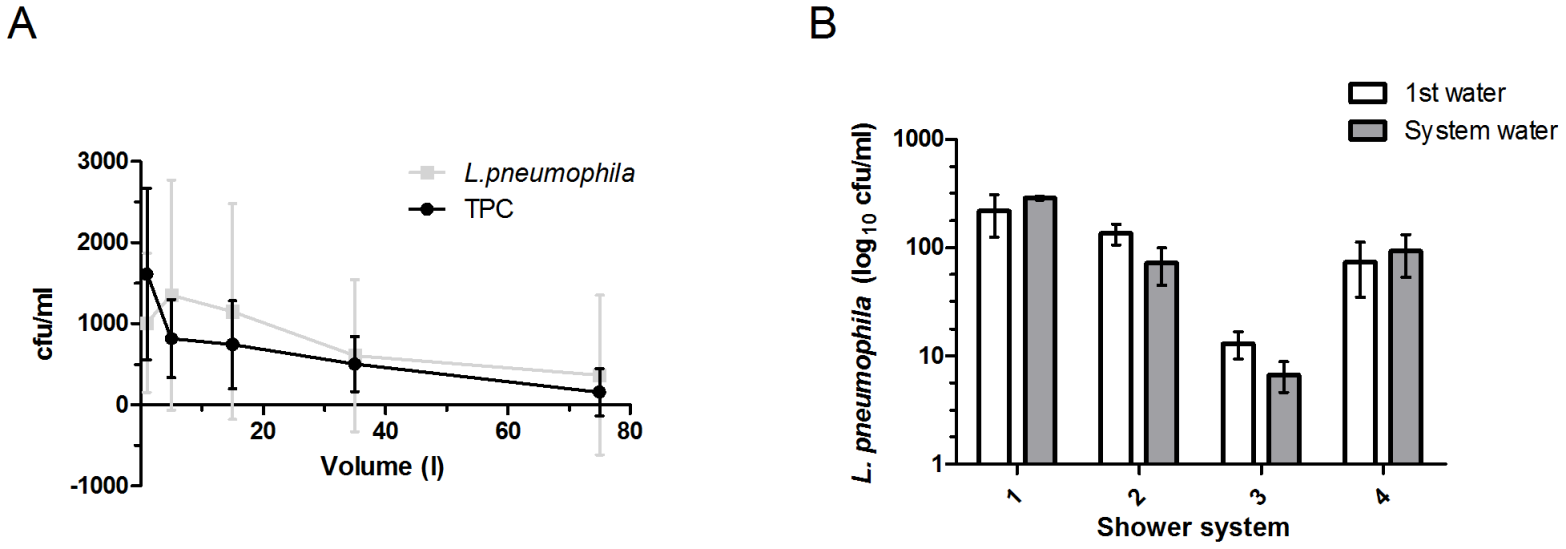
Shower head water compared to system water. A) Concentrations of *L. pneumophila* and TPC of 9 showers at School 1. The sampling volume was 50 ml. The number of samplings per point varied from N = 16 to N = 8. B) Distribution of *L. pneumophila* in different water fractions of 4 shower systems. The white column shows the concentration in the first water collected from the shower head and the grey column the concentration in the system water. There is no statistical difference between the two fractions in any of the systems (nonparametric t-test, P>0.05).

Introducing the new sampling procedure including 50 ml aliquots instead of larger volumes, demonstrated that TPCs were rapidly decimated after turning on the shower (Fig. 2). The concentrations of *L. pneumophila* were comparatively persistent as showers were running (Fig. 3). We observed that a high TPC usually was a shower head phenomenon (Fig. 2, Fig. 3A). By this we mean that high TPCs were primarily demonstrated in the first water. *L. pneumophila*, however, occurred at comparatively stable concentrations in both first and system water (Fig. 3). The high standard deviations (SD) for *L. pneumophila* may be explained by detached pieces of biofilm.

### Longitudinal study of TPC in sports complexes

The 3714 water samples from the shower systems of all municipal sports complexes in Stavanger could not be divided into significant different groups based on TPCs (Scheffé's test). The sports complexes were ranged according to their mean TPC in the period of 2007–2009 (Table 2). At that time, Stavanger municipality assumed that the sports complex systems with the highest TPCs represented the highest risk of being colonized by *L. pneumophila*. As documented in Table 1, that assumption was incorrect. In the period of 2010–2012 sampling from the shower systems of these sports complexes was continued. The sampling was now performed by taking two samples of 50 ml each with a 30 seconds interval. The TPCs of the second water from the period 2010–2012 were comparatively low since they represented system water and not a combination of shower head water and system water as in the period of 2007–2009 (Table 2). The TPCs of the shower head water sampled during 2010–2012 were of the same order of magnitude as the TPCs from 2007–2009, respectively (Table 2). From 2010 and onwards, all samples have been analyzed with respect to both TPC and *L. pneumophila*. *L. pneumophila* was demonstrated in 33% of the 24 sports complexes; 4 cases with sg 1 and 4 cases with sg 2–14. We could not find any pattern supporting that high TPCs indicate presence of *L. pneumophila* (Table 2). In fact, the sports complexes with the highest TPCs were negative regarding *L. pneumophila*. This result is in harmony with the results from School 1 where we found a significant negative correlation between TPC and the concentration of *L. pneumophila* in both first water and system water recently exposed for hot water treatment (Fig. 1).

**Table 2:** Ranging of 24 sports complexes according to their mean Total Plate Count (TPC) from the period 2007–2009.

	2007–2009		2010–2012			
Location	N*	TPC (cfu/ml)	N	TPC FW (cfu/ml)	TPC SW (cfu/ml)	*L. pneumophila* serogroup
Sports complex 1	96	50	7	338	171	
Sports complex 2	168	50	53	465	110	2–14
Sports complex 3	48	90	-	-	-	
Sports complex 4	144	90	10	140	60	2–14
Sports complex 5	145	180	7	862	20	
Sports complex 6	104	220	6	1095	55	
Sports complex 7	92	380	25	1007	880	1
Sports complex 8	145	450	24	2198	294	
Sports complex 9	35	540	18	100	35	
Sports complex 10	46	550	25	445	319	2–14
Sports complex 11	49	600	4	1567	0	
Sports complex 12	158	750	6	506	100	-
Sports complex 13	34	1030	4	1886	733	2–14
Sports complex 14	49	1030	2	550	20	
Sports complex 15	23	1360	2	217	0	
Sports complex 16	100	1510	10	3247	2133	1
Sports complex 17	32	1780	5	3473	1967	
Sports complex 18	206	2110	38	552	137	1
Sports complex 19	108	2230	5	2486	367	
Sports complex 20	331	3690	34	1060	210	1
Sports complex 21	49	3850	4	6920	320	
Sports complex 22	104	4640	12	850	965	
Sports complex 23	55	6490	5	3460	206	
Sports complex 24	35	10770	4	5150	658	

*N: Number of samples.

In this period the standard sampling volume was 250 ml. The ranging is aligned with the sampling of 50 ml aliquots of first water (FW) and system water (SW) in the period 2010–2012. In the period 2010–2012 analyses of both TPC and *L. pneumophila* were done. When present, serogroup affiliation of *L. pneumophila* was determined.

### Water sampling procedure

We demonstrated that high TPCs usually were limited to the first water coming out of the shower head (Fig. 2).

After 2–8 samples, the TPCs stabilized at low levels (Fig. 2). This indicates that the general bacterial concentrations stabilize after approximately 0.5 l.

### Effect of hot water treatment

In the beginning of the present project, TPC was used as an indicator of the effect of both hot water treatment and membrane filters. This decision was based on the assumption that TPC is a valid indicator of the presence of *L. pneumophila*.

#### Nursing homes 1–3

**Figure 4:**
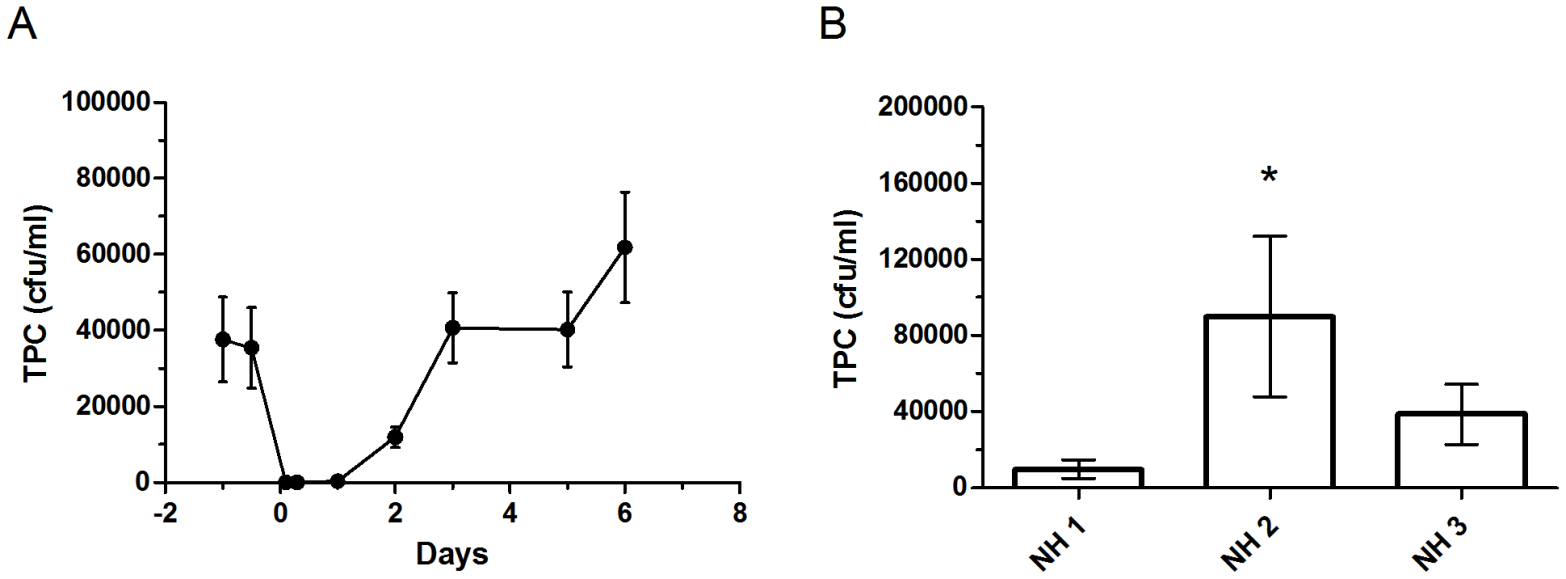
Effect of hot water-treatment on TPC. A) TPC as a function of time before, just after (0), and days after hot water treatment of shower systems (4 different shower system, 6–9 showers per system). The data is presented as an average of all treated shower systems and the error bars represent the standard deviation. B) Comparison of TPC at the three nursing homes NH 1, NH 2 and NH 3. The included showers (N = 8) at NH 1 had not been hot water-treated, while the ones at NH 2 (N = 16) and 3 (N = 14) had been at least monthly treated for years. The TPC was measured one week after thermal treatment. Additionally, at NH 3 the shower armature were systematically treated with a product aimed at eliminating lime depositing. * The difference between NH 1 and NH 2 was significant (P<0.05, non-parametrical).

The water samples were occasionally analyzed with respect to *L. pneumophila* using both the ISO standard procedure and the revised method, but the species was never detected. The results showed that hot water treatment only temporary reduces TPC. Selected showers in the first wing of Nursing Home 1 were hot water-treated. The shower water of the second wing was filtrated while the water of the third wing was not treated at all. For the hot water treated showers at Nursing Home 1, we observed that the TPCs exceeded the pretreatment values three to four days after treatment (Fig. 4A). Comparing both filtrated and non-filtrated shower water from Nursing Home 1 with the two identical nursing homes in which all the showers were regularly hot water treated, confirmed that hot water treatment practically had no effect on TPCs. In fact, the mean TPCs were higher in the at least monthly hot water-treated showers than in non-treated ones (Fig. 4B). In summary, the TPCs were lowest and most stable in the showers not treated at all as compared to hot water-treated showers (Fig. 4B).

#### School 1

During a rehabilitation/vacation period of about three months, *L. pneumophila* sg 2–14 got established in the shower system of School 1. *L. pneumophila* presumably established itself in the two dead-ends left over from the demounting of the system filter just before the respective summer holiday. The presence of *L. pneumophila* made it possible to test the effect of hot water treatment on both TPCs and *L. pneumophila* simultaneously. Our experiments demonstrated that neither *L. pneumophila* nor bacteria in general were considerably long-term inhibited by hot water treatment (Fig. 1). *L. pneumophila* reappeared after 1 to 4 weeks depending on the duration and temperature of the hot water treatment. The period of 4 weeks occurred when the outgoing water from the centralized mixing battery by accident held 55°C for two weeks continuously. As for the selected showers at Nursing Home 1 (Fig. 4A) TPC returned to pretreatment level about four days after treatment. The somewhat longer reestablishment period for *L. pneumophila* may be explained by the comparatively slow growth of both amoebae and *L. pneumophila* [49].

The sports complexes had been monthly or more frequently hot water-treated since 2007 when *L. pneumophila*, both sg 1 and sg 2–14, was demonstrated in the period 2010–2012 (Table 1). This demonstration confirms *per se* that hot water treatment as it was performed is not suitable for preventing the presence of *L. pneumophila* in complex pipeline systems in the long-term.

### Effects of membrane-filtering

**Figure 5:**
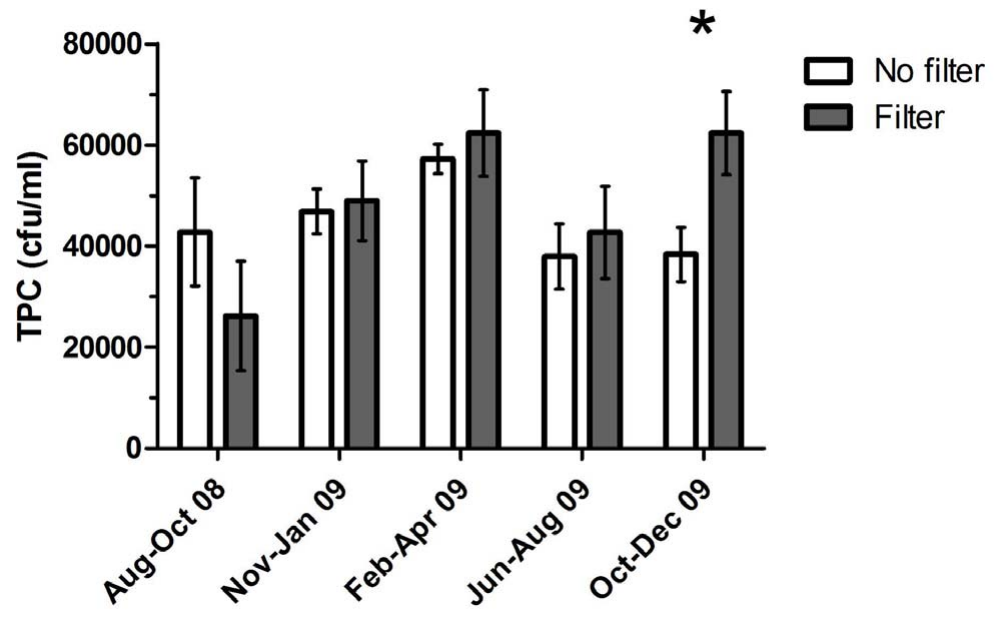
TPC in filtrated versus non-filtrated shower water of Nursing Home 1. The water was filtrated through a membrane filter (0.02 µm) placed in the water flow upstream of the shower rooms in one of three building wings. The * indicates the only sampling point with a statistical difference between filtrated and non-filtrated water (t-test, P<0.05, both parametrically and non-parametrically).

As mentioned, the second wing of Nursing Home 1 was subjected to a membrane filter. The first 3 months of the test period, the TPC appears lower in the filtrated water compared to non-filtrated water, but the difference is not statistically significant. After 15 months, the TPCs became significantly higher in the filtrated water compared to the non-filtrated water (Fig. 5). This may be attributed to increasingly plugging of the filter in spite of the automatic, reverse cleaning process. The plugging was manifested as a significant decrease in water pressure at the downstream side of the filter. Two months after installation, the filter had become so clogged that it was not enough water pressure left to operate the fire hoses. The filter was then taken out of operation for about two weeks, cleaned and reinstalled. In addition to clogging we observed higher bacterial concentrations in filtrated compared to non-filtrated water (Fig. 5). This indicates that the filter did not substantially remove nutrients from the water. Since *L. pneumophila* had never been demonstrated at Nursing Home 1, we are not able to conclude specifically with regard to this species.

As for Nursing Home 1, the TPCs at School 1 were higher downstream than upstream relative to the system filter. The TPC varied somewhat from shower to shower and from time to time (range: below 1000 to above 20 000 cfu/ml). The TPCs of the cold inlet water sampled before and after the rough filter were below the detection limit of 100 cfu/ml during the whole project period. We did not recognize problems with the water pressure during the filtration period at School 1. This may be due to the rough filter at the water inlet to the building.

#### Distal filter in shower head at School 1

Our analyses confirmed that the Shelby antilegionella filter retained bacteria. *L. pneumophila* could not be detected in the shower water despite of the increased sensitivity of the detection method (concentration by filtration; theoretical detection limit: 1 cfu/l, actual detection limit 3–5 cfu/l, data not shown). The absence of detectable *L. pneumophila* in the “Shelby” shower water, presence of *L. pneumophila* in the neighboring showers and high concentration of this bacterium in the drainage water indicate that the Shelby antilegionella filter stops bacteria from passing the filter. During the test period the concentration of *L. pneumophila* was approximately 10 times higher in the drainage water than in non-filtrated shower water. The corresponding TPCs were about 200 times higher. Periodically, the first water of the shower equipped with the Shelby filter showed a comparatively high TPC compared to the first water of the showers without a distal filter (>100x). This may be explained by the extensive filter surface on the downstream side of the filter, sufficient access to nutrients and infrequent use of the shower. The TPC of the “Shelby” shower water decreased as the water was running (data not shown).

The Shelby armature had a manual shower button. It was comparatively demanding to push this button. The shower also turned off very suddenly and loudly, due to the movement of the hydraulic valve within the armature. The water beam from the Shelby shower head was more dispersed and aerosol-forming than the other showers. Interviews with the personnel at School 1 documented that the children seldom if ever used the Shelby shower. The interviewees expressed that the Shelby antilegionella shower armature gave a less comfortable shower experience than ordinary showers, due to mechanical, audio and hydrological differences. Due to the limited use of the Shelby antilegionella shower, we cannot conclude with respect to plugging and robustness of the concept. Additionally, since only one Shelby armature was included in the present study, the reliability of the results is limited (a more extensive study is in progress). The long investigational period with respect to this armature (3 years), however, indicates it is able to withhold the systemic bacteria.

### The surveillance pilot study

**Figure 6:**
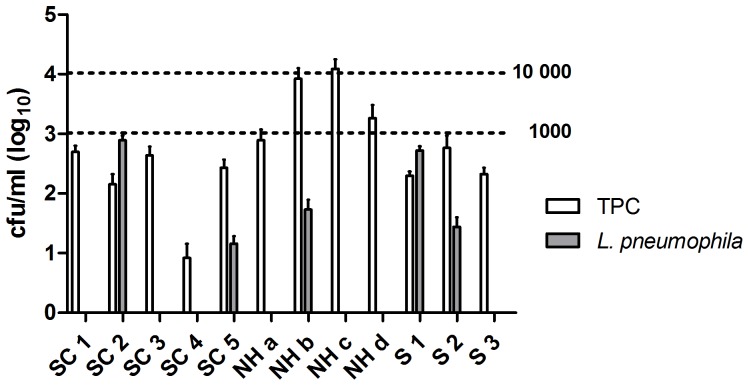
Comparison between TPC and *L. pneumophila* in selected buildings of the surveillance pilot study. SC  =  sports complex, NH  =  nursing home, S =  school. The stippled lines at 1000 and 10000 cfu/ml show the two TPC limits of action with regard to measures against *L. pneumophila* practiced by Stavanger municipality during the first part of the project period.

During the first four years of the project, only *L. pneumophila* sg 2–14 was found. Six of the twenty nine buildings included in the pilot study (i.e. 21%) were positive with respect to this category of *L. pneumophila*. The positive shower systems occurred at two schools, two sports complexes, one nursing home and one preparedness building (fire station). The pilot project confirmed that the TPC limits of 1000 and 10 000 cfu/ml (initially used by Stavanger municipality) are not reliable indicators for the presence of *L. pneumophila* (Fig. 6). Additionally, the on-going surveillance project has demonstrated the presence of sg 1 in several buildings (Table 1). Except for School 1, the showers which tested positive for *L. pneumophila* had been hot water-treated at least monthly for several years at the time of detection.

### Persistence and vulnerability under laboratory conditions

A piece of piping from School 1, a system colonized with *L. pneumophila* sg 2–14, was brought back to the laboratory for further investigation and testing. The biofilm of this piece of piping was barely visible, dry and grainy. Swabbing from this biofilm showed that cultivatable *L. pneumophila* occurred in minority as compared with other kinds of bacterial colonies on the agar surface. This appearance of the biofilm was typical for the biofilm at School 1 during the years it was inspected by pipeline cut-offs. After being filled with cold tap water, *L. pneumophila* soon proliferated to a quite high and stable concentration in the water phase (>4000 cfu/ml) over a period of approximately 12 months. After hot water treatment, *L. pneumophila* did not reappear (tested negative after 143 days).

### From shower head to aerosols

At School 1, we investigated the amount of *L. pneumophila* in the air directly before turning on the showers and after 2 and 5 consecutive pushes at 8 out of 9 showers in the shower room (5 l per push per shower). The shower with Shelby antilegionella filter was not included in this experiment, hence 8 out of 9. Samplings from School 1 were performed 5 times over a period of 10 months (Table 3). The results indicate that there has to be a minimum concentration of *L. pneumophila* in the shower water (>300 cfu/ml) and that the water has to run for a while in order to accumulate a sufficient number of aerosols in the shower room. We were not able to detect any *L. pneumophila* in the aerosols after moderate running (2 pushes) (data not shown). After 5 consecutive pushes (massive running), we measured a concentration of *L. pneumophila* of 2 cfu/m^3^ air in the shower room in 3 of the 5 experiments (Table 3). Due to the sampling volume of air being 0.5 m^3^, only one colony appeared on the agar plate in each of the positive experiments. This means that the result from each individual experiment is of low reliability since only one unit of *L. pneumophila* apparently was dragged into the air sampler. The reproducible results of the experiments, however, indicate that the concentration in air was at the level of 2 cfu/m^3^. The proportion between *L. pneumophila* in water (cfu/ml) as compared to air (cfu/m^3^) was calculated to be between 1.5×10^2^–3.6×10^2^ (Table 3).

**Table 3:** Transfer of *L. pneumophila (L. pn.)* and TPC from shower water to aerosol.

		System water (cfu/ml)		Aerosol (cfu/m^3^)		Transfer factor SW/A
		TPC	*L. pn.*	TPC	*L. pn.*	
Exp 1	System water	2317	715			
	Before showering			392	0	
	After showering			436	2	3.6×10^2^
Exp 2	System water	4283	170			
	Before showering			138	0	
	After showering			340	0	
Exp 3	System water*	525	715			
	Before showering			308	0	
	After showering			434	2	3.6×10^2^
Exp 4	System water*	175	50			
	Before showering			182	0	
	After showering			386	0	
Exp 5	System water*	200	296			
	Before showering			172	0	
	After showering			302	2	1.5×10^2^
Control outdoor air				86	0	

*Between Exp 2 and 3 a routine with daily flushing for 5 minutes was initiated.

Concentrations of *L. pn.* and TPC were measured in system water (SW) and in aerosols (A) and the transfer factor (SW/A) for *L. pn.* was calculated.

Air sampled nearby the outlet of drainage water from the Shelby shower panel did not contain cultivatable *L. pneumophila*.

The laboratory experiments concerning recovery of *L. pneumophila* from air to agar plates showed that the recovery does not seem to depend substantially on the volume of the air sample (Table 4). In most cases there was a slightly higher recovery from 100 l than 500 l at both low and high % RH. This may be due to the mechanical influence of both the air flow and the perforated lid covering the agar plate. Per cent recovery was generally low. The reason for this is that most of the aerosols visibly settled on the surface of the plastic cylinder and the lid. The comparatively low recovery at 95% RH was most probably due to the very high cfu density on the plates as a consequence of high bacterial concentration in the aerosol spray can. In summary, the results indicate that air sampling by use of the SAS sampler is valid (Table 4).

**Table 4:** Recovery of number of cfu of *L. pneumophila* from a spray in the air to GVPC plates as a function of air sampling volume and % RH of the air.

		Per cent bacterial cells recovered	
% RH	Number of bacteria sprayed out	by air volume of 100 l	by air volume of 500 l
20	1.4×10^4^	0.7 (0.5, 0.7, 0.9)*	0.3 (0.04, 0.3, 0.5)*
95	8.6×10^5^	0.08 (0.06, 0.08, 0.09)*	0.11 (0.06, 0.08, 0.2)*

*The 3 replicates in parenthesis.

### Perception and performance of hot water treatment - Interviews and observations

Based on the interviews with municipal operational personnel, hot water treatment could be divided into three sequential parts, i.e. (i) running the hot water, (ii) the interval between two runs, and (iii) water sampling after running. The operational personnel expressed a similar and clear comprehension of how to perform the hot water running *per se*. They admitted, however, that it was difficult to maintain 65°C distally in the pipelines for 5 minutes. Some of them compensated for this by breaking the treatment down into stages. The operational personnel differed substantially with respect to time interval between two treatments. Some were performing the treatments with regular intervals of about four weeks. Others performed the treatment at a different time each month. Also the time interval between hot water treatment and subsequent sampling differed substantially both with respect to the individual operator as well as between them. Some sampled at more or less a fixed time after treatment while others did sample at varying intervals. The sampling varied from days to weeks after treatment. The personnel differentiated between “good” and “bad” samples. The good samples were those with TPCs below 10 000 cfu/ml because then extra treatments were not needed. Obtaining good samples became a goal in itself. The showers were often run for some minutes before the sample was taken since this gave “better” samples. One of our interviewees consequently sampled cold water into a sampling bottle which was placed on the floor beneath the shower. By this, the sample consisted of cold water which had been running for quite a while.

The interviews revealed that the operational personnel did not follow the water sampling protocol. As a consequence, from the beginning of 2009 all sampling and bacterial analyses were performed by the researchers in the project. By this standardization, the effect of thermal treatment was from 2009 and onwards tested as specified in the protocol and not by individual adaptations.

### The commercial suppliers

The providers of antilegionella products communicated according to what has been designated the Propaganda Model [50]. This kind of communication is characterized by lack of interest in the other party's views. It is used to exploit for profit rather than to enlighten. The providers of products used the Norwegian legislation and the Norwegian *Legionella* guidance [19] as “alibi” when arguing for their product. Neither the law nor the guidelines, however, impose any specific measures upon the municipalities when it comes to shower systems. The providers presented their products nearly as a guarantee against *Legionella*. They emphasized positive characteristics by their respective product without presenting scientific documentation. When the municipality asked for such documentation, the providers promised to forward it. None of the documents that were forwarded represented scientific documentation. Most of them had the form of one-page diplomas or insignificant certificates.

### Anxiety – A driving force

The grounded theory approach involves open, axial and selective coding [46]. The concepts of scant knowledge, uncertainty and anxiety emerged from the open and axial coding. The selective coding revealed a rather scant knowledge about bacteria in general among the stakeholders. This scant knowledge is transmitted to the understanding of *L. pneumophila* and the respective risk. Additionally there is a real, scientific uncertainty about how to handle *L. pneumophila* [1], [2], [3]. The combination of scant knowledge and uncertainties made stakeholders think about *Legionella* in ways that evoked anxiety. An example is parents of children at School 1 who expressed uncertainty and associated anxiety when informed about the presence of *L. pneumophila* at “their” school. When shown an agar plate seeded with *L. pneumophila* they literally jumped backwards. The agar plate was passed around and the parents were informed about serogroup affiliation (sg 2–14), virulence and lack of any known case infected with these serogroups in Norwegian showers. The parents then appeared relieved. Governmental and municipal representatives expressed anxiety of being held accountable should anyone be infected from a water source within their premises. Our observations and non-standardized interviews in meetings and seminars indicated that it was more important to demonstrate that something was done than that this “something” had an effect. In the standardized interviews, the operational personnel expressed a similar attitude. They were not afraid of being harmed themselves, but of being blamed if anyone should be infected from “their” showers. This attitude was supported by the fact that they did not use safety equipment when performing hot water treatment and sampling.

As described in the present study, *L. pneumophila* has been confirmed in several municipal shower systems. While the anxiety associated with *Legionella* constitutes a threat for some stakeholders, it represents an opportunity for others. For example, businesses selling water cleaning technology and laboratories selling microbial analyses position themselves to benefit from a significant marked expansion when *Legionella* is reported.

In summary, anxiety seems to have been a considerable driving force in the Norwegian risk governance of *L. pneumophila* in municipal shower systems. Further, this anxiety seems to be based on insufficient knowledge among stakeholders and real uncertainties concerning the risk associated with *L. pneumophila*.

## Discussion

Due to the uncertainties about prevention measures, infection sources, virulence and sampling methods concerning *Legionella* [1], [2], [3], it is difficult to get an overview regarding the risk of being infected with *L. pneumophila* in general and from showers in particular.

Until 2012, the Norwegian guidelines for prevention of *Legionella* infection [18], [19], [20] did not emphasize that water samples from public showers should include analysis of *Legionella per se*. The assumption was that high TPCs indicate the presence of a massive biofilm and thereby the presence of *L. pneumophila*. We did not find any significant positive correlation between concentration of *L. pneumophila* and TPC. It should be emphasized that in the investigated systems with relatively high TPCs (>5×10^4^ cfu/ml), *L. pneumophila* was never detected. After hot water treatment, there was a low, but significant negative correlation between concentration of *L. pneumophila* and TPC. Since the significant negative correlation only applied to the period after treatment and the fact that the correlation was weak, low TPCs are not considered a valid indicator of *L. pneumophila*. The conclusion is that TPC represents an invalid indicator of presence and/or concentration of *L. pneumophila* in shower systems. This conclusion is in harmony with previous findings [40], [51], [52]. In the third and latest edition of the Norwegian guidelines for prevention of *Legionella* infection [20], it is recommended to use *Legionella* spp. as an indicator of *L. pneumophila* sg 1. Since *L. pneumophila* has recently been found to account for 96.5% of all reported cases of Legionnaires' disease in Europe and sg 1account for 86.2% of the total of 96.5% [9], there should be no a need for indicators concerning Legionnaires' disease. The most valid and convenient method with respect to showers, should be to analyze for *L. pneumophila* directly, both by qPCR and cultivation, systematically followed by serogrouping and sequence-based typing (SBT).

The standard method ISO 11731∶1998(E) [38] for detection and enumeration of *Legionella* is rather time-consuming and thereby expensive. This laboriousness is probably part of the rationale behind the use of high TPCs as an indicator of *Legionella*. We evaluate the revised method, the dripping method, as valid for detection and enumeration of *L. pneumophila* in shower water. The arguments are that (i) we use the same agar media as in the standard ISO 11731∶1998(E) [38], (ii) the filtration or centrifugation steps, which has been shown to reduce the reliability of the bacterial counts [47], [48], are omitted, and (iii) the theoretical mathematical detection limit of 5 cfu/ml is evaluated as satisfactory since there is no established action threshold with respect to concentration of *Legionella*. The varying recovery from the concentration steps of the original method reduces its reproducibility and thereby its reliability. The dripping method has by nature a higher reliability since the bacteria are very gentle handled during the whole procedure. Our revised method is similar to a previously published method designated “the direct inoculum method” [40]. The authors' conclusion was that the direct inoculum method provided counts comparatively near the real numbers of legionellae present in the samples. Due to the opportunistic pathogenic nature of *L. pneumophila* [4], [14], [53], a general dose-response relationship cannot be established. The risk of being infected is more a function of the host's immune system than the infectious dose. The “European Working Group for Legionella Infections” [21], however, has recommended a theoretical mathematical detection limit for *Legionella* bacteria of less than or equal to 0.1 cfu/ml in water samples. We don't find any arguments supporting this low detection limit when it comes to shower systems. Schoen and Ashbolt [32] developed a model which predicted the proportion between *Legionella* in shower water and air in a risk perspective. High prediction was based on a *Legionella* concentration of 3.5×10^5^ cfu/ml in water which corresponded to a concentration of 3.5×10^3^ cfu/m^3^ in air. The proportional relation of 10^2^ is of the same size as found in the present project (1.5–3.6×10^2^). The concentrations of *L. pneumophila* in the shower systems of Stavanger municipality, however, were much lower than 3.5×10^5^ cfu/ml. According to Schoen and Ashbolt [32], the referred concentrations were meant to indicate critical concentrations that might lead to potential infection. The model, however, does not seem to have taken into account the opportunistic nature of *L. pneumophila*. The Norwegian Institute of Public Health has in its latest edition of the Norwegian guidelines for prevention of *Legionella* infection [20] changed the limit of 0.1 cfu/ml [19] to an unspecified requirement of reporting of the actual concentration of *L. pneumophila* in cases where it has been confirmed. On basis of the above argumentation, a sensitivity of 5 cfu/ml should be satisfactory concerning *L. pneumophila* in shower water.

In the environment, *Legionella* are found in both a cultivatable and a viable but not cultivatable (VBNC) state [54], [55]. Studies have shown that as little as 0.1–1% of the bacteria in a sample can be cultured, and that stress like heat or chlorination can trigger cultivatable *Legionella* to enter the VBNC state [54], [55], [56], [57], [58], [59], [60]. In the VBNC state, the bacteria do not replicate in culture media. Interestingly, uptake and passage through amoeba in the laboratory can stimulate VBNC *Legionella* to re-enter the cultivatable state [61], [62], [63]. Therefore, relying exclusively on cultivation techniques to evaluate treatment effectiveness and assurance of a *Legionella*-free -system may therefore cause false conclusions. However, it is assumed that these bacteria do not initiate disease when present in the VBNC state [61], [64], [65]. Therefore, monitoring the viable bacteria seems satisfactory as a routine environmental surveillance in a public health perspective.

In Norway the recommendation for thermal treatment is 70°C for a minimum of 5 minutes [20]. WHO recommends thermal treatment at 70°C for 30 minutes to eradicate *Legionella* [66], [67]. Studies have shown that even this treatment does not necessarily kill all *Legionella* cells, but merely causes them to enter the VBNC state [56], [66], [67]. In an experiment where samples were analyzed after 5, 10, 20, 30 and 60 minutes of thermal treatment at 70°C, there was a gradual transition from viable and cultivatable *Legionella,* via the VBNC stage to dead bacteria [56]. In addition, environmental *Legionella* strains have been shown to differ in heat tolerance and that repeated thermal treatment over a longer period can select for thermally tolerant/resistant strains [56], [67]. Farhat el al. [67] concluded in their study that heat shock treatment cannot be used as curative treatment for *Legionella*. This is consistent with our findings. Although the thermal disinfection appeared successful under laboratory conditions, the bacterial concentrations of the investigated systems are only temporarily reduced following hot water treatment. In addition, the literature also reports conflicting results regarding the success of thermal treatment [68], [69], [70], [71], [72]. This further supports our findings that it is not straight-forward to use thermal control as a long-term preventive measure against *Legionella* in complex systems. Whether the reappearance of *L. pneumophila* is due to regrowth of persistent bacteria or to resuscitation of VBNC as suggested by Allegra et al. [66], the effect is short term. It is neither desirable nor practical for the municipality to carry out thermal treatment of their systems with the necessary frequency to ensure no detection of cultivatable *Legionella* bacteria. This applies both with regards to the development of heat-tolerant bacteria as described by Allegra et al. [66] and for the cost in time and money for operating personnel to conduct the thermal treatment. Adding that several of the municipal systems have difficulties reaching the conditions recommended by the Norwegian regulation (70°C, 5 minutes), we conclude that thermal treatment as a long-term preventive measure for controlling *Legionella* in complex systems did not work as intended.

In fact, none of the measures tested in the present study eliminated or permanently reduced *a priori* established *L. pneumophila* from complex pipeline systems. The distally mounted membrane filter, however, seemed to stop *L. pneumophila* from reaching the shower heads and thereby the user. Since *L. pneumophila* had not been found in the buildings before the system filters were installed, we are not able to conclude specifically on their retention of *Legionella*. On the assumption that bacteria do not pass through membrane filters, *L. pneumophila* cells can only occur at the down-stream side of the filter if they were present before the filter was installed. However, such a guarantee can hardly be given if the pipelines have been exposed to water before installation of the filter. Concerning chemical treatment to control *L. pneumophila*, it should be evaluated if the risk concerning environment and health is higher than the risk related to the bacteria themselves, with focus on both short and long-term effects [3]. This topic has not been sufficiently focused upon when it comes to *Legionella* [3].

Based on research literature as outlined by Wiik and Boccadoro [3] and the present project, we are not able to identify any universal measure for buildings with complex water pipeline systems except for the distal filter, as effective when it comes to lowering the risk of acquiring Legionnaires' disease from showering. This conclusion is in harmony with the fact that none of the commercial suppliers were able to display research-based documentation on the effect of their products. The lack of scientific documentation also concerned products that we chose not to test due to their potential long- term harmful effect on health and environment. An example of such a product is the combination of silver and copper ions [3].

Our results indicate that stagnant water due to dead ends and/or scant use of the shower system increases the risk of establishment of *L. pneumophila*. Dead-ends are assumed to promote formation of biofilm containing *L. pneumophila* [73], [74], [75], [76], [77]. It has also been found that *L. pneumophila* can establish itself in the water phase independently of biofilm formation [78], [79]. The research literature, however, differs with respect to the significance of water movement for the establishment of *L. pneumophila*. In small-scale plants, Liu et al. [80] and Waines [30] were not able to demonstrate such significance. The gap between findings may be grounded on different environmental or experimental conditions. Our conclusion so far is that regular water flow and as few dead ends as possible do not seem to promote the establishment of *L. pneumophila*.

It may be questioned if municipal showers or showers in general represent a substantial risk as compared to other man-made water systems. The community-acquired outbreaks of Legionnaires' disease in Norway have been exclusively linked to cooling towers and air scrubbers, i.e. outdoors systems [20]. Our results indicate that *L. pneumophila* is only scantly present in the air even after massive showering with water having high concentrations of this species. This finding may be partly explained by the high relative humidity (RH) (≈100%) in shower rooms when showers are running. At high RH, aerosols or droplets stay large and becomes even larger, which imply that they are falling comparatively fast to the floor, i.e. outside the breathing zone [33]. In fact, showering seems to be of marginal importance when it comes to Legionnaires' disease, a statement supported by Stout [2]. In a survey of private bathroom outlets (shower heads or taps), Borella et al. [81] reported that the risk of acquiring pneumonia was not significantly larger in legionellae-positive homes than in legionellae-negative ones. Interestingly, Borella et al. [81] reported that *Legionella* spp. were detected in 22.6% of the private homes. In our surveillance pilot study we found that 21% of the municipal buildings were positive for *L. pneumophila*. The limited infection risk from showering is in harmony with the recently published surveillance results from ECDC [9] which reports that only in 6.3% of 331 positive environmental findings isolates could be matched to community-acquired clinical findings. It was not indicated in the report that these 6.3% originated from showers.

The relationship between *Legionella* in water and the amount disseminated via aerosols depends on factors as degree of aerosol production, air humidity and temperature [82]. In Norway, mean monthly outdoor RH is relatively stable throughout the year [83], i.e. around 80%. The temperatures, however, are higher in summer than in winter time. Due to comparatively high outdoor temperatures, the saturation deficit of the air is higher in summer than in winter [84]. This means that the aerosols from outdoor water systems will be smaller in summer time due to evaporation, which implies higher probability of being inhaled [84]. This corresponds to the fact that outbreaks of Legionnaires' disease in Norway have occurred during summer time. The outbreaks could exclusively be traced to outdoor water systems as cooling towers and air scrubbers [20]. It is relevant that cooling towers are primarily run in summer time. It is also relevant that there have been no outbreaks of Legionnaires' disease in Norway after implementation of new restrictions regarding cooling towers in 2008 [85]. In Europe, about 60% of all cases of Legionnaires' disease occur in the warm season, i.e. from June to October [9]. The association between weather conditions and occurrence of legionellosis has been studied in the Greater Philadelphia Metropolitan Area [29] and Switzerland [86]. The conclusion from both studies was that the occurrence of community-acquired Legionnaires' disease was best predicted by humid and warm weather. The authors did not mention seasonality regarding running of cooling towers. Neither did they discuss their results in the perspective of aerosol sizes.

The Norwegian legislation gives guidelines about how to handle different water systems with respect to *Legionella*. One of the regulations orders monthly microbial sampling from cooling towers, air scrubbers, humidifiers, and indoor fountains [85]. Neither private nor public showers are included in this list. The regulations are further outlined in the Norwegian *Legionella* guidance [20]. This guidance mainly gives advices of how things can be done, not clear requirements. In spite of the legislation not specifying regular sampling from municipal showers, sampling from such systems receives substantial attention in the *Legionella* guidance. This ambiguity seems to be made the most of by providers of antilegionella products. In meetings with the municipality and at conferences, these actors gave the impression that sampling from public shower systems are demanded by law. According to our observation studies, the reference to law represented a strong indication of *L. pneumophila* as a substantial threat when it comes to showers. The commercial actors most often argued in an asymmetric, one-sided way. This way of communication [50] is shown to be more persuasive than two-sided argumentation towards people with low familiarity with the issue at hand [87]. The majority of the observed stakeholders had only superficial knowledge about bacteria in general and *Legionella* in particular. This lack of knowledge, combined with a lacking best practice regarding safe handling of *Legionella* in complex shower systems, have contributed to the implementation of measures that seem to be of low validity in a risk governance perspective. The combination of technological and psychological perspectives on risk is in accordance with prevailing risk governance theory [88], which emphasize that not only the technological but also the psychological perspective substantially influences what the stakeholders choose to do in order to reduce the risk. As an example, it was the interviews and observations which documented that scant knowledge, uncertainty, ambiguity and anxiety affected the planning and implementation of the measures against *L. pneumophila*.

Only a few variants of *L. pneumophila* represent a documented risk with respect to Legionnaires' disease [5], [6], [9], [11], [13]. Both sg 1 and sg 2–14 have been confirmed in our municipal shower systems. There have been no reported cases of Legionnaires' disease associated with municipal shower systems in Norway since 2007. This supports our evaluation that the risk of acquiring Legionnaires' disease from such systems seems low for the general public. A further support, is that the interviewed, operational personnel had never acquired Legionnaires' disease in spite of not using safety equipment during hot water treatments. We evaluate surveillance as the most convenient measure when it comes to *L. pneumophila*. By this we will know which buildings are positive for *L. pneumophila*. According to Stout and Yu [89], a high risk building will be turned into a low risk one by surveillance. The argument is that patients with indications of Legionnaires' disease can get a quicker diagnosis and treatment if we know that the patient has been exposed to a contaminated shower system.

In conclusion, it seems difficult to eliminate *L. pneumophila* once it has been established in a complex shower system with branches, long stretches, varying temperatures etc. Based on the above discussion, however, the presence of low concentrations of *L. pneumophila* strains in municipal shower systems does not seem to represent a general threat to human health. In cases of immunocompromized individuals, however, distal membrane filters represent a solution. We have only tested measures evaluated as safe, i.e. measures that do not represent a higher risk to human health than *L. pneumophila* itself. In our opinion, the absence of effect is not discouraging in the perspective that most strains of *L. pneumophila* found in municipal shower systems do not seem to represent a significant public health risk under most circumstances. We evaluate surveillance as the most convenient risk reducing measure when it comes to *L. pneumophila* in municipal shower systems. Knowledge about *L. pneumophila* and ability to handle its presence make the overall risk low and acceptable.
